# Sunflower Oil and Cholesterol Nanoemulsion: A Novel Carrier for Micafungin to Combat Multi-Resistant *Candida auris*

**DOI:** 10.3390/pathogens13070549

**Published:** 2024-06-28

**Authors:** Gabriel Davi Marena, Alejandro López, Gabriela Corrêa Carvalho, María del Pilar Marín, María Dolores Pérez Ruiz, Jose Manuel Pérez-Royo, María Ángeles Tormo-Mas, Patricia Bernabé, Eulogio Valentín, Taís Maria Bauab, Marlus Chorilli, Javier Pemán, Alba Ruiz-Gaitán

**Affiliations:** 1 Severe Infection Research Group, Health Research Institute La Fe, 46026 Valencia, Spain; gabriel.marena@unesp.br (G.D.M.); alejandro_lopez@iislafe.es (A.L.); manuel_perez@iislafe.es (J.M.P.-R.); eulogio.valentin@uv.es (E.V.);; 2 Department of Drugs and Medicines, School of Pharmaceutical Sciences, São Paulo State University (UNESP), Araraquara 14800-903, SP, Brazil; gabriela.correa@unesp.br (G.C.C.); marlus.chorilli@unesp.br (M.C.); 3 Department of Biological Sciences, School of Pharmaceutical Sciences, São Paulo State University (UNESP), Araraquara 14800-903, SP, Brazil; tais.bauab@unesp.br; 4 Cell Biology Unit, Health Research Institute La Fe, 46026 Valencia, Spain; pili_marin@iislfe.es; 5 Department of Pathological Anatomy, La Fe Hospital, 46026 Valencia, Spain; perez_mdorui@gva.es; 6 Department of Microbiology and Ecology, University of Valencia, 46010 Valencia, Spain; 7 Department of Medical Microbiology, University and Polytechnic La Fe Hospital, 46026 Valencia, Spain

**Keywords:** *Candida auris*, nanoemulsion, micafungin, *Galleria mellonella*, emerging infections

## Abstract

*Candida auris* is an emerging, multidrug-resistant yeast that causes systemic infections, mainly in hospitalized or immunosuppressed patients. This pathogen has a high mortality and morbidity rate. This study aims to evaluate the antifungal potential of micafungin (MICA) encapsulated in a nanoemulsion (NEM) against four clades of *C. auris* and other non-*C. auris* species. The antifungal potential of MICA and NEM was evaluated by determining mature biofilm inhibition (0.78–50 µg/mL). The antifungal activities of MICA and NEM (5.92 mg/Kg) were evaluated using an in vivo model of *Galleria mellonella*. The results showed that NEM intensified the antibiofilm action of MICA, especially in 48 h mature biofilms. In vivo results displayed a higher effectiveness of NEM against all clades of *C. auris* tested, inhibiting the fungal load in the hemolymph and tissues of *G. mellonella* with a difference of 3 log10. In addition, *C. auris* infection caused granulomas surrounded by hemocytes, mainly at the lower and upper ends. Conversely, *C. albicans* developed pseudohyphae, biofilms, filaments, and chlamydospores. In conclusion, encapsulation of MICA in a nanoemulsion enhances its antifungal activity against mature biofilms of *C. auris*. This strategy may be considered a therapeutic approach for the control of infections and the dissemination of this new global health threat.

## 1. Introduction

In 2019, the Centers for Disease Control and Prevention (CDC) estimated that the number of annual cases of disease and deaths from resistant microorganisms would be 2,600,000 and 44,000, respectively. The same year, the CDC included *Candida auris* as an urgent threat due to its potential ability to be resistant to the three main classes of antifungals: azoles, polyenes and echinocandins [1]. The mortality rate for an invasive *C. auris* infection can be as high as 60% [2]. According to Wang et al. [3] the 90% of *C. auris* isolates are resistant to fluconazole. Furthermore, due to the COVID-19 pandemic, intensive care units were overloaded and, consequently, the number of cases of co-infection with *C. auris* increased [4].

Given the increase in cases of *C. auris* and limited therapies, nanomedicine has become a hope for the development of new therapeutic models that can contribute to the reduction in new cases and improve the performance of available antifungals. Nanoemulsions (NEs), which are emulsions formed by water and oil with the addition of surfactants, are one of the most investigated nanotechnological models. NEs are kinetically stable nanoparticles composed of droplets smaller than 500 nm [5,6,7]. Another study considered NEs as an excellent drug delivery system because they provide controlled and selective release guaranteeing greater drug safety from a preclinical point of view [8].

Several studies have described that NEs contributed to increasing the antifungal potential of substances against pathogenic fungi. Shahid et al. [9] reported that a cationic NE loaded with ketoconazole can be considered a promising therapy for greater permeation and therapeutic efficacy; Jawaid et al. [10] observed better antimicrobial behavior, including anti-*C. albicans*, for an NE loaded with citronella essential oil, and Marena et al. [11] noticed that encapsulated amphotericin B in an NE was significantly better against a mature biofilms of *C. auris* CDC B11905.

Therefore, this study aimed to evaluate the in vivo and in vitro antifungal efficacy of micafungin-encapsulated nanoemulsions against different clades of *C. auris* and non-*C. auris* yeasts.

## 2. Materials and Methods

### 2.1. Development and Characterization of the Nanoemulsion

NE was formulated according to the method described by Marena et al. [6,11]. The formulation consisted of 10% polyoxyethylene (20) cetyl ether (Brij^®^ 58, Sigma Aldrich, Steinheim, Germany), and soy phosphatidylcholine (Lipoid, Ludwigshafen, Germany) in a 2:1 ratio, 10% sunflower oil (Essential Engineering, São Paulo, Brazil) and cholesterol (Sigma Aldrich, Steinheim, Germany), and 80% phosphate-buffered saline.

### 2.2. Fungal Strains

For the in vitro and in vivo assays, the following strains were used: *C. auris* VPCI479/P13 (India, Clade I—InP13), *C. auris* AL1 (CLADO I), *C. auris* CBS10913 (Japan, Clade II—JAP 1), *C. auris* Kro 2 (Clade II), *C. auris* CBS 15603 (Spain, Clade III—SP96), *C. auris* (Spain, Clade III—SP94), *C. auris* VEN C6, *C. auris* BRA 2 (Venezuelan, Clade IV), *C. albicans* ATCC—5314—and *Candida parapsilosis* ATCC—22019.

### 2.3. Evaluation of Antibiofilm Efficacy

Assessment of the antibiofilm activity of MICA and NEM was performed in two stages: (i) pre-adherent antibiofilm and (ii) mature antibiofilm, as described by Marena et al. [11] with some modifications. The controls used in each assay were as follows: growth control (inoculum+YEPD), NE control (inoculum+YEPD+NE), YEPD sterility control (YEPD only), MICA sterile control (YEPD+MICA), and NEM sterile control (YEPD+NEM). Initially, 100 µL of inoculum (10^6^ cells/mL) suspended in PBS was transferred to 96-well microplates and incubated at 37 °C for 2 h for cell pre-adhesion. Then, plates were washed with 200 µL of PBS to remove non-adherent yeast. For the pre-adherent biofilm assay, yeasts adhered to microplates were treated with 100 µL of MICA or NEM (0.07–20 µg/mL) solubilized in Yeast Extract Peptone Dextrose Broth (YEPD, Scharlab S.L., Barcelona, Spain) and incubated at 37 °C for 24 h. For the mature biofilm assay, 100 µL of YEPD was added to each well and incubated at 37 °C for 48 h. Then, mature biofilms were washed with 200 µL of PBS and treated with 100 µL of MICA or NEM solubilized in YEPD (concentration range of 0.78–50 µg/mL). 2,3-bis(2-methoxy-4-nitro-5-sulfophenyl)-5-[carbonyl(phenylamino)]-2H-tetrazoliumhydroxide (XTT^®^ at 0.005 g/10 mL—Thermo Fisher Scientific, Waltham, MA, USA) was added to each well, incubated for 2 h at 37 °C followed by spectrophotometric reading at 492 nm in order to determine the metabolic activity of the biofilm.

### 2.4. Confocal Laser Scanning Microscopy

For pre-adhesion, 1 mL of PBS solution containing 1 × 10^6^ cells/mL was transferred into a 24-well microplate containing a sterile crystal (1 cm in diameter) and incubated at 37 °C for 2 h.

After the pre-adhesion time, the non-adhered cells were removed by washing with PBS (1 mL), keeping only the adhered cells on the crystal surface. After washing, the wells were filled with YEPD (1 mL) and incubated for 48 h at 37 °C. Then, the biofilm formed was washed with PBS (1 mL), followed by treatment with 1 mL of the prepared samples and diluted in YEPD (25 µg/mL). After, biofilms were incubated at 37 °C for 24 h. Next, the biofilm was washed again with Ringer (1 mL) three times and then stained with a live/dead stain (LIVE/DEAD^®^ Yeast Viability Kit, Thermo Fisher Scientific) according to the manufacturer’s instructions. A solution containing FUN (1:1000) in a ringer was added (500 µL/well), and biofilm was incubated for 30 min at 30 °C. After, the biofilm was washed with Ringer (1 mL/well) and finally, crystal coverslips were mounted using Mowiol. Microscopy and imaging were performed with a Leica SP5 confocal microscope (Leica, Wetzlar, Germany) using the sequential mode and a 40× oil objective. The excitation wavelength was 488 nm for FUN 1, and the emission wavelength was 530 nm. For image capture, three visual fields were randomly selected for each sample and observed in triplicate. Each experiment was repeated at least three times.

#### 2.4.1. In Vivo Antifungal Activity

For this assay, only one isolate of *C. auris* was selected from each clade, namely InP13 (Clade I), JAP 1 (Clade II), SP96 (Clade III), and VEN C6 (Clade IV). *C. albicans* ATCC-5134 and *C. parapsilosis* ATCC-22019 were used as controls.

The in vivo assay using the *G. mellonella* model was performed as described by Garcia-Bustos et al. [12] with some modifications. Larvae were used, weighing 250–350 mg. On the infection day, 10^4^ and 10^5^ cells/larva inoculum were used for *C. auris* strains and non-*C. auris* strains, respectively (concentrations of non-*C. auris* species were higher because previous trials showed that lower concentrations of inoculum were not able to cause infection in *G. mellonella*). Larvae were immobilized by placing them on ice for 2–3 min. Before inoculation, the larval prolegs were sterilized with a swab soaked in 70% ethanol. A standardized inoculum consisting of 10 µL PBS + Ampicillin (PBS+AmP, Sigma Aldrich, Steinheim, Germany) at a concentration of 20 µg/mL was injected into the penultimate proleg of each larva using a Hamilton Microliter™ syringe. After 2 h, 10 µL of MICA or NEM (solubilized in PBS+AmP until reaching a concentration of 5.92 mg/Kg, 5 × MIC_90_) was injected into the penultimate right proleg of each larva (20 larvae/group). Controls were as follows: (i) infection control group (infected larvae+PBS+AmP), (ii) larvae without infection (larvae+PBS+AmP), and (iii) NE control (infected larvae+10 µL NE). All larvae were incubated at 37 °C for 24 h. The treatment was performed every 24 h for 5 days.

After 24 h of incubation, three larvae from each group were selected for total hemolymph extraction via decapitation, followed by tissue collection. Hemolymph samples were collected in Eppendorf tubes, diluted in PBS + Ampicillin (20 μg/mL) at ratios of 1:2, 1:10, or 1:100, and cultured on Sabouraud–chloramphenicol agar (SDA, PanReac AppliChem, Barcelona, Spain). The tissue samples were placed into tubes containing 2 mL of PBS + Ampicillin, homogenized using an Ultra Turrax^®^ T25 (Janke & Kunkel IKA^®^, Staufen, Germany), and cultured on SDA following dilution in PBS + Ampicillin at ratios of 1:10, 1:100, and 1:1000. Subsequently, 100 μL of hemolymph and tissue homogenates was cultured on SDA and incubated at 37 °C for 48 h, followed by colony-forming unit (CFU) quantification, expressed as CFU/mL of hemolymph and CFU/mL of homogenized tissue. Additionally, three larvae from each group were sacrificed after 2 h of incubation to culture their hemolymph and tissues, determining the CFU count immediately post-infection (time zero). A two-way analysis of variance (ANOVA) was used to compare the difference between the treated group (MICA and NEM) and the untreated group (NE and PBS+AmP). Statistical analysis was also used to compare the differences between the MICA-treated and NEM-treated groups. Tukey’s post hoc test was performed for multiple comparisons between the groups. A value of *p* < 0.05 was considered significant.

#### 2.4.2. Histopathology

The infection and treatment were performed as described above. Tissue fixation and staining were performed as described by Garcia Bustos et al. [12] with some modifications. The larvae were collected 120 h after infection and treatment, anaesthetized with 5% ethanol, and transferred into tubes containing 10 mL of 4% formalin for tissue fixation and preservation. After 20 days, the larvae were processed using sagittal cuts, fixed in paraffin, and stained with hematoxylin–eosin (HE) and periodic acid–Schiff (PAS). Histological analysis was performed using an optical microscope (Carl Zeiss, Jena, Germany, Axiolab ^®^E).

## 3. Results

### 3.1. Development and Characterization

The NEs (NE and NEM) were developed and characterized in previous assays and, according to the results, they presented an average hydrodynamic size of around 40 nm, good uniformity, electronegative charge, and good stability for three months. Cryogenic scanning electron microscopy tests showed NEs with spherical particles. Finally, NEs were shown to be non-toxic in alternative in vivo tests using *G. mellonella* [6,11].

### 3.2. Pre-Adherent Antibiofilm Activity

**Figure 1:**
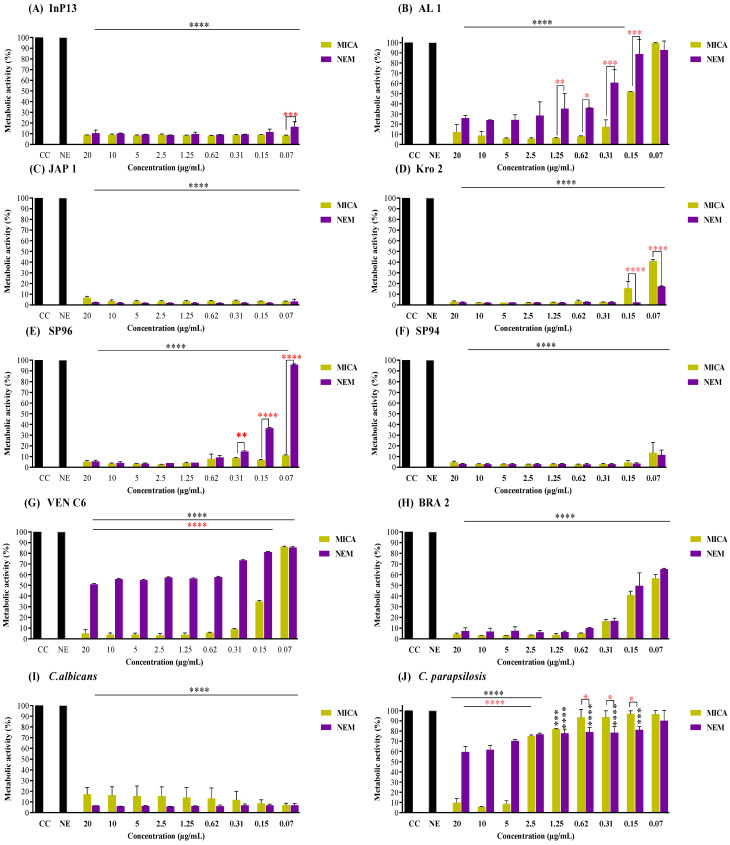
Pre-adherent *Candida* spp. biofilms treated with MICA and NEM. MICA: micafungin; NEM: nanoemulsion + micafungin; NE: nanoemulsion; GC: growth control. (****): Statistical difference between the treated group (MICA and NEM) and the untreated group (GC and NE). Asterisk in red indicates difference between treated groups. Black asterisk indicates difference with the infection control group. (*) *p* = 0.05; (**) *p* = 0.005; (***) *p* = 0.0005; and (****) *p* < 0.0001.

According to the results in the Supplementary Materials Table S1, MICA presented an MIC_90_ against *C. auris* isolates ranging from 0.15 to 5 µg/mL (MIC_50_ of 0.0542 to 0.9610 µg/mL), while NEM was >5 µg/mL (MIC_50_ of >5 to 1.491 µg/mL). Figure 1 shows the antibiofilm activity of MICA and NEM, indicating that the most susceptible strains to both treatments were InP13 (Clade I), JAP 1 and Kro (Clade II), SP94 (Clade III), and *C. albicans* (Figure 1A,C,D,F,I, respectively). The strain that was least susceptible to treatment was *C. parapsilosis* (Figure 1J).

NEM was least effective against AL 1, SP96, and VENC6 (Figure 1B,E,G, respectively). The inhibition of the metabolic activity of biofilms treated with MICA and NEM at 0.15 µg/mL was 48.1 and 11.1% (*p* = 0.0009) for AL 1, 93.2% and 63.5% (*p* < 0.0001) for SP96, and 65% and 18.9% (*p* < 0.0001) for VEN C6, respectively. However, NEM exhibited a higher efficacy at lower concentrations, decreasing the metabolic activity of Kro and *C. parapsilosis* (Figure 1D,J, respectively). At a concentration of 0.15, NEM treatment resulted in the metabolic inhibition of pre-adherent biofilms at rates of 97.91% for Kro (*p* < 0.0001) and 18.9% for *C. parapsilosis* (*p* = 0.0251). Under the same conditions, MICA promoted an inhibition of metabolic activity of 84.3% and 3% for Kro and *C. parapsilosis*, respectively.

**Figure 2:**
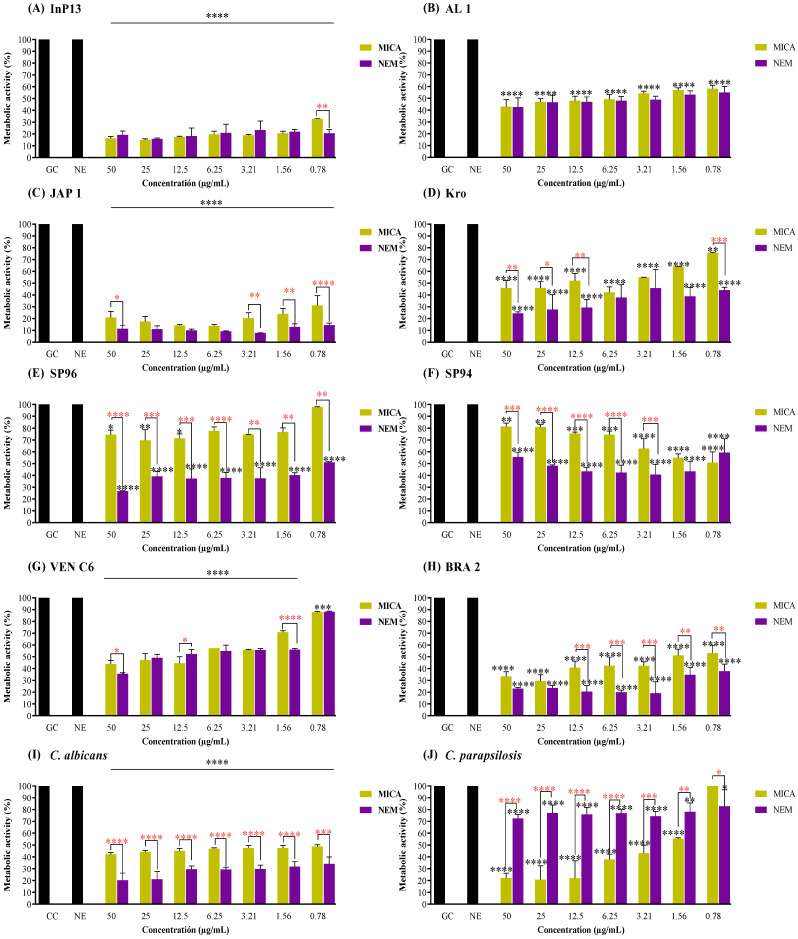
Mature biofilm of *Candida* spp. treated with MICA and NEM. MICA: micafungin; NEM: nanoemulsion + micafungin; NE: nanoemulsion; GC: growth control. (****): Statistical difference comparing the treated group (MICA and NEM) with the untreated group (GC and NE). Asterisk in red indicates difference between treated groups. Black asterisk indicates difference with the infection control group; (*) *p* = 0.05; (**) *p* = 0.005; (***) *p* = 0.0005 and (****) *p* < 0.0001.

Figure 2 shows the metabolic activity of the mature biofilms treated with MICA and NEM. Notably, NE improved the antibiofilm potential of MICA, being the most effective treatment against all strains, except for AL1 and *C. parapsilosis* (Figure 2B,J, respectively). NE maintained the antifungal activity of MICA even at lower concentrations. For example, at a concentration of 1.56 µg/mL, there were no differences between MICA and NEM against the mature biofilm of Clade I.

In comparison, Clade II NEM-treated biofilms (at 1.56 µg/mL) exhibited a substantial metabolic inhibition of 87.2% (*p* = 0.003) and 61.2% (*p* = 0.002) for JAP 1 and Kro, respectively. In comparison MICA, treatment resulted in a metabolic inhibition of 76.1% and 36.13% for JAP 1 and Kro, respectively. A noteworthy statistical difference was observed in Clade III biofilms, where treatment with NEM (1.56 µg/mL) was more effective, with metabolic inhibition of 59.9% (*p* = 0.006) and 56.5% (*p* = 0.089, no difference) against SP96 and SP94, respectively. Metabolic inhibition was lower when treated with MICA with 23.3% and 45.1% of inhibition for SP96 and SP94, respectively.

The Clade IV mature biofilm exhibited an enhanced susceptibility to NEM treatment at a concentration of 1.56 µg/mL, resulting in metabolic inhibition of 43.9 (*p* < 0.0001) and 65.4% (*p* < 0.004) for VEN C6 and BRA 2, respectively. Post MICA exposure, VEN C6 and BRA 2 exhibited a metabolic inhibition of 29.2% and 48.8%, respectively.

Among non-*C. auris* strains, NEM demonstrated greater statistical efficacy against mature *C. albicans* biofilms, with 68.3% metabolic activity inhibition compared to 52.4% with MICA treatment (*p* < 0.0001) at 1.56 µg/mL. Conversely, mature *C. parapsilosis* biofilms exhibited heightened susceptibility to MICA treatment, particularly at 1.56 µg/mL, with 44.6% metabolic inhibition (*p* = 0.004), whereas NEM showed lower effectiveness, resulting in a 21.9% inhibition of metabolic activity.

### 3.3. Confocal Laser Microscopy

To confirm the antibiofilm metabolic activity findings, a representative strain from each clade, along with a non-*C. auris* (*C. albicans*) strain, was carefully selected to assess the effects of the respective treatments on mature biofilms. Confocal microscopy was employed to evaluate both metabolic activity and biofilm thickness, providing a comprehensive perspective on the distribution and state of cells within the *Z*-axis of the biofilm structure.

**Figure 3:**
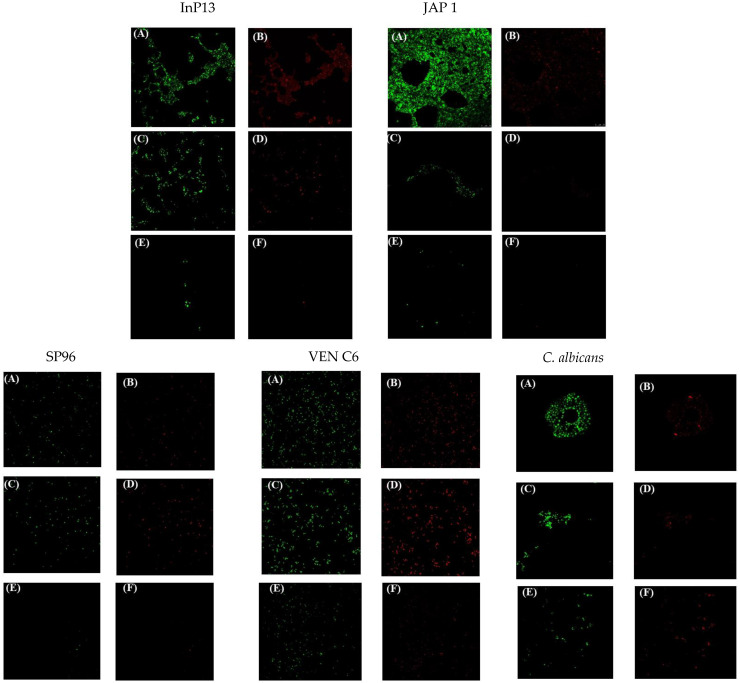
Metabolic activity by confocal microscopy analysis of *C. auris* biofilms treated with MICA and NEM. (**A**,**B**) Control; (**C**,**D**) biofilms treated with micafungin; (**E**,**F**) biofilms treated with nanoemulsion + micafungin.

The microscopic images in Figure 3 showed distinct results in metabolic activity and biofilm structure. In Figure 3A,B, aggregated cells were observed in all biofilm control groups. In contrast, MICA treatment produced an altered biofilm state characterized by single cells (Figure 3C,D), an effect that was more evident with NEM treatment with a predominance of single cells (Figure 3E,F). A significant contrast in metabolic activity was observed between the NEM and MICA treatments. As shown in Figure 3F, NEM treatment resulted in a reduction in metabolic activity due to a decrease in the biofilm.

Regarding biofilm thickness, the *Z*-axis cell density consistently showed higher values in control biofilms compared to treated ones. InP13 and JAP 1 strains exhibited a similar decrease in biofilm thickness after being treated with NEM and MICA. In particular, the InP13 control had a thickness of 9.5 μm, which decreased to 5 μm after both treatments. Similarly, JAP 1 had a thickness of 9.5 μm in the control group and 6 μm in both the NEM and MICA treatments, with no significant differences observed.

However, a notable decrease in the thickness of the biofilm was observed in the NEM-treated biofilms compared to the MICA-treated biofilms for the SP96 and VENC6 strains. Specifically, the biofilm thickness of the SP96 control was measured at 7.95 μm, which decreased to 5.94 μm and 3.83 μm for MICA and NEM treatments, respectively. Likewise, the VENC6 biofilm control had a thickness of 9.6 μm, compared to 9.4 μm for MICA and 5.92 μm for NEM treatments. Consistent results were obtained for *C. albicans*, with NEM treatment resulting in a greater reduction in biofilm thickness (5.92 μm) than MICA treatment (9.4 μm) and the control (9.6 μm).

### 3.4. In Vivo Antifungal Activity

**Figure 4:**
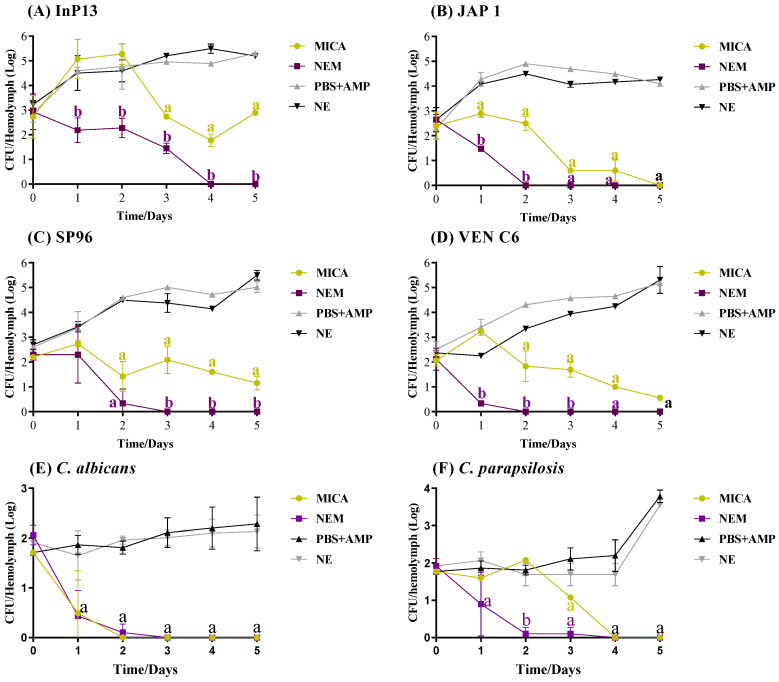

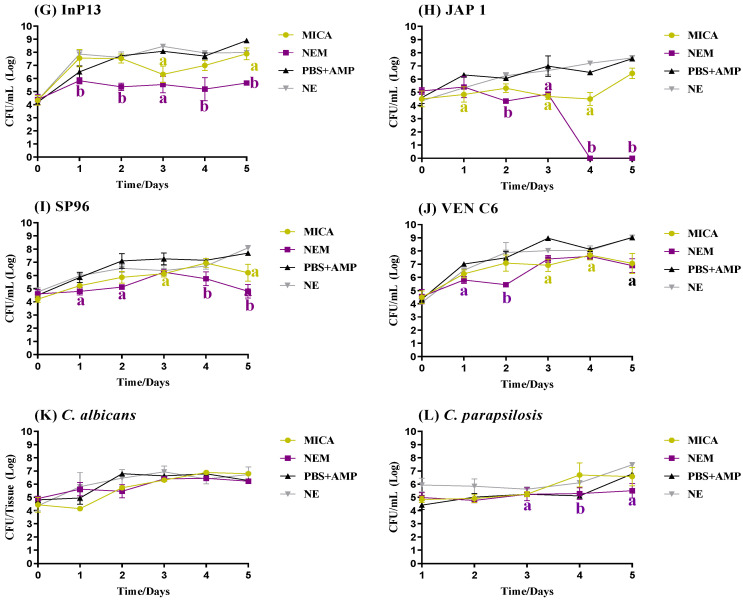
Antifungal activity of MICA and NEM against *C. auris* and non-*C. auris* in *G. mellonella* hemolymph and tissues. (**A**–**F**): Hemolymph; (**G**–**L**): tissue; NE: nanoemulsion; PBS+AmP: basic phosphate solution + ampicillin (20 µg/mL); MICA: micafungin; NEM: nanoemulsion + micafungin; absence of letters: no statistical difference; letter (a): the difference between infection groups (NE and PBS+AmP) with treated groups (MICA and NEM); letter (b): difference with the infection group and difference with MICA.

The results of the antifungal activity of MICA and NEM in the hemolymph are shown in Figure 4A–F. Both treatments were statistically significant (*p* = 0.05, *p* < 0.0001), reducing the fungal load in hemolymph compared to the infection group (NE and PBS+AmP). When comparing the treatment options, NEM showed superior antifungal activity against all strains tested, except for *C. albicans* (Figure 4E), where both treatments were similar. NEM completely inhibited the fungal load in hemolymph after 2, 3, and 4 days of treatment in *G. mellonella* infected with JAP 1, VEN C6, SP96, *C. albicans*, InP13, and *C. parapsilosis* (*p* < 0.0001). However, despite the significant results, the infection persisted in the groups treated with MICA. Complete inhibition of the fungal load was achieved only in the group infected with *C. albicans*, *C. parapsilosis,* and JAP 1 after 3, 4, and 5 days of treatment, respectively. This represents only 50% of the strains (25% for *C. auris*), as shown in Figure 4B,E,F. There is a clear difference between the strains of *C. auris*, especially on the second day; e.g., on the second day, the fungal load in of hemolymph for the group treated with MICA and NEM was 10^5^ and 10^2^ CFC/mL for InP13 (*p* = 0.029), 10^2^ and 0 CFC/mL for JAP 1 (*p* = 0.028), 10^1.4^ and 10^0.3^ CFC/mL for SP96 (*p* < 0.0001), and 10^1.8^ and 0 CFC/mL for VEN C6 (*p* < 0.0001), respectively.

Figure 4G–L show the fungal load in tissues of infected *G. mellonella*, and as can be observed, the tissue obtained a greater amount of yeast than the hemolymph for all strains. Furthermore, NEM demonstrated superior antifungal activity when compared to MICA, suggesting that NE increases the action of MICA against infection, except for *C. albicans* (Figure 4K), where no difference between treatments was observed. After five days of treatment, the fungal load for the MICA and NEM groups infected with InP13 was 10^7.9^ and 10^5.6^ CFU/mL, respectively. According to Figure 4G, NEM was significantly more efficient (*p* = 0.0003). The group infected with JAP 1 had a fungal burden of 10^6.4^ CFU/mL after five days of MICA treatment. However, NEM eliminated the fungal burden after four days and was statistically efficient (*p* < 0.0001, Figure 4H). Furthermore, there was also a significant difference (*p* = 0.0018) between MICA and NEM treatment after five days in the SP96-infected group, with counts of 10^6.2^ and 10^4.8^ CFU/mL, respectively (Figure 4I). After five days of treatment, MICA reduced the fungal burden for half of the strains tested (InP13, SP96, and VEN C6), as shown in Figure 4G,I,J, compared to the infection control. On the other hand, NEM was more effective in inhibiting the fungal load against all strains, except for *C. albicans*, after the same duration of treatment.

### 3.5. Histopathology

**Figure 5:**
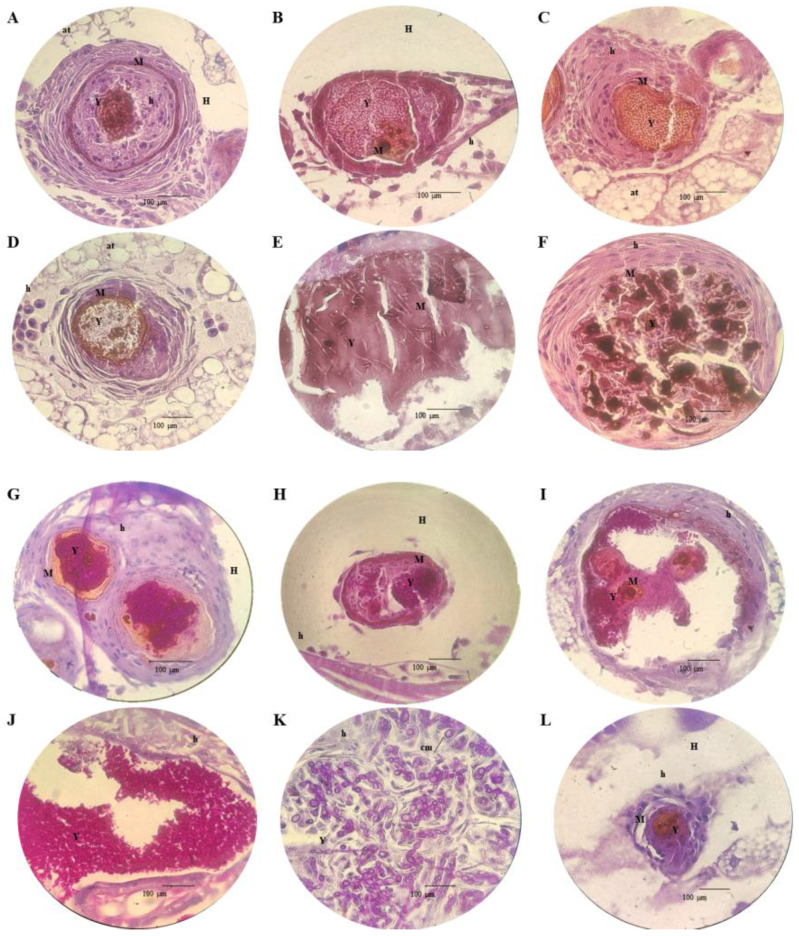
Histopathological findings in *G. mellonella* tissues infected with *C. auris* and non-*C. auris* and stained with PAS and HE (×100). Hematoxylin–eosin: (**A**) InP13; (**B**) JAP 1; (**C**) SP96; (**D**) VEN C6; (**E**) *C. albicans*; (**F**) *C. parapsilosis*. Periodic acid–Schiff: (**G**) InP13; (**H**) JAP 1; (**I**) SP96; (**J**) VEN C6; (**K**) *C. albicans*; (**L**) phosphate buffer + *C. parapsilosis*.

Figure 5 displays the histopathological responses of *G. mellonella* to fungal infection. The images show clusters of yeasts (Y) involved in an intense immunological response caused by hemocytes (h) and forming granulomas. The granulomas showed an increase in melanin (M) caused by the infection. Most granulomas were found in the upper extremities (head and thorax) and the lower extremities (tail and lower intestine). The granulomas were found to be surrounded by adipose tissue (at), dispersed in hemolymph (h), adhered to the wall of organelles, close to the cuticle (ct), or in muscle tissue (mt). There is a large infiltration of yeasts with pseudo-hyphae inside the organelles (intestine), with a strong level of melanization and infection.

Figure 5G–L display the PAS-stained tissue of *G. mellonella* after infection. The presence of a layer of agglomerated yeasts is observed (Y) within granulomas or biofilm. There was a striking difference in the mechanism of infection between *C. auris* and *C. albicans* strains. *C. auris* produced granulomas of various sizes, mainly on the two extremities of the larvae. *C. albicans* showed an increased ability to invade tissues, pseudohyphae formation, and chlamydospores (cm).

## 4. Discussion

During the 20th century, polyenes, azoles, and flucytosine were the main types of antifungal treatment available. However, their administration carried the risk of nephrotoxicity and hepatotoxicity. Widespread use, combined with a limited number of options, has led to resistance in many fungal strains. In view of this, the use of combination therapy has increased in recent years in order to control the disease and combat resistance. Studies to address this problem have led to the development of innovative drugs that mark a turning point in the 21st century. Echinocandins, specifically micafungin, caspofungin, and anidulafungin, have emerged as a new class of antifungal agents that inhibit β-(1,3) d-glucan synthase in the fungal cell wall, resulting in morphological deformation, osmotic lysis, and fungal death. Echinocandins are widely accepted for treating systemic *Candida* infections; however, reports indicate that up to 5% of *C. auris* strains in the United States rapidly acquire resistance during treatment [13,14,15,16].

Recent years have seen a notable focus on developing nanoscale-controlled drug delivery systems: nanomedicine. This technology improves pharmaceutical agent performance in the human body, enhancing selectivity, bioavailability, absorption, and interactions with pathogens, while minimizing adverse side effects, as indicated by recent studies [6,17,18]. Characterized by nanoscale droplet formation in immiscible liquids like water and oil, these colloidal dispersions, known as NEs, are considered nanosystems providing significant drug protection against environmental factors (pH changes, microbial enzymes, and immune responses), ultimately improving bioavailability and selectivity [6,17].

In response to the global threat of *C. auris* infection and the potential benefits of drug encapsulation in nanoemulsions (NEs), this study evaluated the performance of MICA encapsulated in an NE against four *C. auris* clades. The results from planktonic cell assays showed no detectable antifungal activity for NEM. In contrast, MICA exhibited an MIC range of 0.09 to 5 µg/mL (Supplementary Materials). Similarly, a previous study reported no activity of a MICA-loaded NE against the *C. auris* strain CDC B11903. This study hypothesizes that NE may reduce the antifungal efficacy of MICA by slowing drug release, which may promote microbial growth [19].

Biofilms, consisting of a community of microorganisms enveloped by a protective polymer matrix, impede drug penetration and protect internal cells from immune responses [20]. Recent studies indicate that biofilms evade host immune defenses [21,22], which is crucial in medical settings where *Candida* species form biofilms on devices and cause systemic infections with a mortality rate of 30% [22]. The *C. auris* species has the ability to adhere differently to surfaces, develop biofilms, and resist antifungal therapy [23]. While MICA exhibits good antifungal activity against *Candida* spp. biofilms [24,25], our study evaluates the potential of MICA encapsulated in an NE, demonstrating superior antibiofilm activity against *C. albicans* and different *C. auris* clades.

NEM was not as effective against preformed biofilms when compared to MICA. Furthermore, it is noted that there was a significant increase in metabolic activity at low concentrations, which may be related to ineffective doses (low inhibition of metabolic activity). However, NEM was significantly better against mature biofilms. The improved antibiofilm activity of encapsulated MICA may be due to the NE interacting with the polymeric matrix of the biofilm, allowing nanoparticle fusion and protein denaturation with better penetration and delivery of MICA among the yeast community within the biofilm. NE transports greater amounts of MICA within biofilms, allowing direct contact, destabilizing yeast cell membranes, and causing greater metabolic inhibition than conventional drugs. Conventional drugs have greater difficulty penetrate the polymer matrix, which acts as a barrier to antifungal activity [26,27].

Regarding biofilms, the polymeric matrix makes it difficult for drugs to penetrate inside, limiting antifungal therapy. According to Lee et al. [28], the dilution of antifungal molecules results in the persistence of the infection (less drugs present). Taking into account that NEs have the ability to better penetrate the polymeric matrix [27], it is believed that the NE increased the amount of MICA within mature biofilms, which provided a greater inhibition of metabolic activity. However, notes that MICA was more effective against pre-formed biofilms (except for Kro and *C. parapsilosis* in lower concentrations). A possible justification would be the small amount of polysaccharide matrix present, which did not hinder MICA penetration, inactivating biofilm formation with greater efficiency. The lesser action of NEM may be related to a controlled or sustained release of MICA by NE, as described by Singh et al. [5] about the advantages of an NE.

Other studies have reported similar results, such as Giongo et al. [29], who reported better antifungal activity of compounds encapsulated in an NE against *Candida* spp. biofilms. The authors developed an NE containing geranium oil, and the NE was more efficient against biofilms of *C. albicans*, *C. tropicalis*, and *C. glabrata* compared to free oil. Marena et al. [11] used an NE to encapsulate amphotericin B against a mature biofilm of *C. auris* CDC B11903; their results indicated that the NE improved the antibiofilm potential of amphotericin B, particularly at low concentrations. Junqueira et al. [30] evaluated the response of an NE loaded with zinc 2,9,16,23-tetrakis(phenylthio)-29H,31H-phthalocyanine (ZnPc) with photodynamic therapy against *Candida* spp. biofilms and other emerging pathogens, and the therapy was effective in reducing the number of cells in the biofilms formed.

Although the resistance against MICA is lower when compared to other antifungal classes (reaching 5%), three mechanisms are of concern. Mutations in the FKS gene, responsible for encoding the fks subunit, encode a different subunit, resulting in non-inactivation by echinocandins and the normal production of glucans in the cell wall. Another resistance mechanism is the presence of a molecular chaperone, Hsp90, responsible for reducing fungal stress in response to antifungals and increasing cellular integrity. Finally, fungi can increase the production of mannans, another important component of the cell wall, in response to glucan depletion, increasing the integrity of the fungal wall (resistance known as the salvage response) [28].

In vivo models, such as mice and rats, for example, are extremely important in the investigation of new antimicrobials before they are used in humans; however, high costs and ethical considerations limit their use. *G. mellonella* larvae have become one of the most used alternative in vivo models nowadays in the evaluation of antimicrobial potentials. This model is appropriate for evaluating the effectiveness of new antifungal substances against yeasts and filamentous organisms [31]. Furthermore, its functional similarity in the immune response to infection and that observed in the immune response in mammalian organisms make this model even more suitable for investigating new potent antimicrobials. However, as in other in vivo models, *G. mellonella* has some disadvantages. Among the limitations, the larvae do not present an adaptive response and the results obtained need to be confirmed in other models, in order to increase the reliability of the data [32]. In view of this, the antifungal potential of NEM and MICA was evaluated in a model of infected *G. mellonella* and, according to the results, NE potentiated the action of MICA against all strains when compared to the group treated with MICA. Although in in vitro results, the NE intensified the antifungal action of MICA against mature biofilms, this difference was not seen for all strains, such as InP13 for example (Figure 2A). A possible justification is that while in mature biofilm the treatment period was 24 h, in vivo the treatment time was 5 days, making it possible to observe the therapeutic difference over time.

Another important characteristic is that, although NEM was statistically more efficient than MICA, the therapeutic behavior was different. While the fungal load of JAP 1 and VEN C6 (Clade II and IV, respectively) was completely inhibited in hemolymph after two days of treatment with NEM, it was only possible to inhibit 100% of InP13 after 4 days of treatment. *C. auris* is a haploid and diploid yeast, where some clades differ in aspects such as virulence rate, growth, and global gene expression profiles. Diploid cells, for example, have a slower growth rate; however, they are considered more virulent than haploid cells in a systemic infection model in mice [33]. Therefore, it is believed that the different therapeutic profiles between the strains are due to intraspecies heterogenicity, providing different virulence and growth rates during systemic infection in *G. mellonella*.

Finally, our histological results show the formation of granulomas resulting from hemocyte displacement around infection, in order to combat the progression of the infection. to. Furthermore, it was observed that granulomas are located more frequently between the extremities, with a high level of melanin. The group infected with *C. albicans* showed a higher profile of tissue infection, with pseudohyphae formation, filamentation, tissue infiltration, chlamydospores, and biofilm formation. According to one study, chlamydospores are related to the persistence of the infection as they are a mechanism of resistance and survival [34]. *C. auris* species elicits a host immune response leading to more granuloma formation than *C. albicans* and *C. parapsilosis*, particularly in non-aggregating strains. Garcia et al. [12] observed heterogeneity among species of *C. auris* species regarding pathogenicity. The authors found that non-aggregative strains were more pathogenic, while no significant difference in melanization levels was established. Additionally, according to the authors, histological assays showed that *C. auris* infections mimicked those observed for *C. albicans*, with effective dissemination from the early stages of infection, the presence of filamentation, and the formation of pseudohyphae. On the other hand, Muñoz et al. [35] evaluated the infection profile of *C. auris* Ca432 and Ca 386 and, according to the authors, the formation of hyphae/pseudohyphae was not observed and the yeasts were dispersed in the tissues.

Although the results are of great relevance in the control of a systemic infection caused by a multidrug-resistant fungus, it is important to highlight three limiting factors. The first limitation is the small quantity of clinical isolates used since the diversity of *C. auris* in the world is considerably high. Another factor is the low number of larvae used per group (*n* = 20), which could be increased and strengthen the conclusions found. Finally, the failure to carry out an experiment on mammals would bring greater reliability to the results.

## 5. Conclusions

This study underscores the importance of exploring innovative drug delivery systems, such as micafungin-loaded nanoemulsions, that exhibit a high antibiofilm potential against all clades of *C. auris* and *C. albicans*. These results were confirmed by in vivo studies using the *G. mellonella* model and have significant implications for the treatment of drug-resistant strains such as *C. auris*. Nanomedicine contributes valuable insights into the potential to enhance antifungal efficacy and highlights the complexities of host–pathogen interactions in the context of different *Candida* species.

## Data Availability

Data are contained within the article.

## Supplementary Materials

The following supporting information can be downloaded at: https://www.mdpi.com/article/10.3390/pathogens13070549/s1. Table S1: Determination of the minimum inhibitory concentration of MICA and NEM against *Candida* ssp.
