# Supplementary material for: Sunflower Oil and Cholesterol Nanoemulsion: A Novel Carrier for Micafungin to Combat Multi-Resistant Candida auris
Source: Pathogens. 2024 Jun 28;13(7):549. doi: 10.3390/pathogens13070549 (PMC11279427; doi:10.3390/pathogens13070549)
Supplement: Supplementary file 1 [file pathogens-13-00549-s001.zip › pathogens-3046707-supplementary.pdf]

Material and Methods

Determination of the minimum inhibitory concentration (MIC)

The MICA and NEM were solubilized in Roswell Park Memorial Institute (RPMI) 1640 medium (Sigma Aldrich, Steinheim, North Rhine-Westphalia, Germany) and evaluated at 0.003-5 µg/mL. Growth (inoculum+RPMI), NE (inoculum+RPMI+NE), and RPMI sterility (RPMI only) were used as controls. Briefly, 96-well microplates containing $1x10^3$ cells/mL treated with MICA or NEM, were incubated at 37ºC for 48h. After incubation, MIC_{50} and MIC_{90} values (MICs at which 50% and 90% of the isolates were inhibited, respectively) were calculated.

All plates were measured spectrophotometrically, and each experiment was performed at least three times on three different days. To calculate the MIC_{50} and MIC_{90}, the absorbance data from the three tests were analyzed using GraphPad Prism 8.0 software

Table S1. Determination of the minimum inhibitory concentration of MICA and NEM against *Candida* ssp.

	**Minimum Inhibitory Concentration** (µg/mL)																			
	CLADE I				CLADE II				CLADE III				CLADE IV				No-*C. auris*			
	InP13		AL 1		JAP 1		Kro		SP96		SP94		VEN C6		BRA 2		Ca 5314		*C.pa 22019*	
	MIC_{90}	MIC_{50}	MIC_{90}	MIC_{50}	MIC_{90}	MIC_{50}	MIC_{90}	MIC_{50}	MIC_{90}	MIC_{50}	MIC_{90}	MIC_{50}	MIC_{90}	MIC_{50}	MIC_{90}	MIC_{50}	MIC_{90}	MIC_{50}	MIC_{90}	MIC_{50}
MICA	0.62	0.0542	1.25	0.1071	0.31	0.0223	0.15	0.079	5	0.9610	5	0.1010	1.25	0.0860	5	0.2365	0.09	0.006	1	0.1290
NEM	>5	>5	>5	>5	>5	>5	>5	>5	>5	2.5642	>5	2.5642	>5	1.491	>5	1.491	0.09	<0.006	>5	>5
NE	-	-	-	-	-	-	-	-	-	-	-	-	-	-	-	-	-	-	-	-

Table S1. MICA: Micafungin; NEM: Nanoemulsion + Micafungin; NE: Nanoemulsion; -: yeast growth; Ca 5314*: Candida albicans* 5314; Cpa 22019: *Candida parapsilosis* 22019.
